# Sensing nucleotide composition in virus RNA

**DOI:** 10.1042/BSR20230372

**Published:** 2023-09-05

**Authors:** Raymon Lo, Daniel Gonçalves-Carneiro

**Affiliations:** Imperial College London, Department of Infectious Disease, Imperial College London, London, U.K.

**Keywords:** antiviral, Coding Bias, host-virus interaction, Nucleotide, Virus

## Abstract

Nucleotide composition plays a crucial role in the structure, function and recognition of RNA molecules. During infection, virus RNA is exposed to multiple endogenous proteins that detect local or global compositional biases and interfere with virus replication. Recent advancements in RNA:protein mapping technologies have enabled the identification of general RNA-binding preferences in the human proteome at basal level and in the context of virus infection. In this review, we explore how cellular proteins recognise nucleotide composition in virus RNA and the impact these interactions have on virus replication. Protein-binding G-rich and C-rich sequences are common examples of how host factors detect and limit infection, and, in contrast, viruses may have evolved to purge their genomes from such motifs. We also give examples of how human RNA-binding proteins inhibit virus replication, not only by destabilising virus RNA, but also by interfering with viral protein translation and genome encapsidation. Understanding the interplay between cellular proteins and virus RNA composition can provide insights into host–virus interactions and uncover potential targets for antiviral strategies.

## Introduction

A fundamental property of nucleic acids is the ability to store and transmit information via the sequence of nucleotides they contain. Coding sequences are composed of an array of codons that are translated by ribosomes to produce polypeptides. This flow of information is universal and can be found both in living organisms and the viruses that infect them. Due to the degeneracy of the genetic code, with 61 codons coding for 20 amino acids, organisms can develop and sustain coding biases. These biases can manifest as an overrepresentation or scarcity of specific codons, codon pairs or nucleotide combinations in protein-coding sequences [1]. Such biases can influence gene expression, by impacting mRNA stability or translation efficiency, or even evade detection by the immune system. As a result, viruses are constrained by host-induced pressures on coding sequences, and some viruses may have nucleotide compositions that have been selected to optimise replication in specific hosts [2].

Nucleotide composition refers to the relative proportions of the four nucleotides that make up DNA or RNA molecules, i.e. adenine, guanine, cytosine and thymine/uridine. Additionally, composition of nucleic acids may also refer to the overall GC content – which is the percentage of guanine and cytosine bases in a given sequence – or to the frequency of certain dinucleotides. At the most basic level, nucleotide composition affects the physical and chemical properties of the nucleic acid molecule, such as its melting temperature [3], stability [4], bendability [5] and the propensity to form complex secondary and tertiary structures [6]. For example, melting temperatures of DNA molecules increase with GC-content while G-rich RNA sequences have the tendency to form a stable secondary structure known as G-quadruplex (G4). Nucleotide composition also affects mutagenesis of nucleic acids after insults such as oxidation [7] and UV-light damage [8]. Beyond its effects on physicochemical properties of nucleic acids, nucleotide composition governs many aspects of cell biology, primarily by modulating how proteins interact with nucleic acids. Some of these interactions are conserved in eukaryotes over millions of years of evolution and they are important for many biological functions.

Viruses, as obligate intracellular pathogens, are exposed to thousands of proteins in cells that recognise nucleotide composition. Viruses are under increased selective pressure since they (1) must hijack cellular machinery to replicate while (2) avoiding recognition by the immune system but (3) maintaining recognition of their own nucleic acids to ensure genome packaging. In this review, we focused on how various cellular proteins recognise nucleotide composition in virus RNA, and how advancements in RNA:protein mapping technologies have enabled the identification of RNA-binding profiles for many RNA-binding proteins (RBPs). Mostly we focused on proteins whose activity leads to reduced virus replication, however, as discussed, many proteins inhibit infection of one virus but aid the replication of others. Canonical virus RNA sensors, such as retinoic acid-inducible gene I (RIG-I)-like receptors (RLRs) and some Toll-like receptors (TLRs) display certain compositional biases in the types of molecules they recognise; e.g., RIG-I preferentially binds U-rich sequences with interspersed cytosine nucleotides [9], while TLR7 preferentially binds to uridine-containing sequences [10]. However, since these receptors primarily recognise double-stranded RNA or differentially localised single-stranded RNA, and their properties have been reviewed elsewhere [11], in this review we will focus on emerging evidence for other cellular proteins that recognise nucleotide composition in virus RNA.

## RNA-binding preferences of human proteins

Over the past decades, the development of novel techniques to determine with high precision what sequence motifs RBPs recognise have significantly change our understanding of molecular biology. One such approach is crosslinking and immunoprecipitation (CLIP) coupled with RNA sequencing [12]. CLIP methods, such as PAR-CLIP [13], HITS-CLIP [14] and iCLIP [15], combine UV cross-linking of RNA–protein complexes with immunoprecipitation. UV cross-linking helps to covalently link the RBP to the bound RNA molecules, preserving their interactions even after stringent washing steps. After immunoprecipitation, cross-linked RNA:RBP complexes are purified, and the RNA sequences are sequenced to identify binding sites. Another approach to determine RNA-binding preferences is systematic evolution of ligands by exponential enrichment (SELEX) [16,17]. SELEX is an iterative process used to identify RNA sequences that bind to a specific RBP. It involves generating a large pool of random RNA sequences and subjecting them to multiple rounds of selection and amplification. The RNA sequences that bind to the protein of interest are enriched with each round, and through subsequent sequencing and analysis, the binding motifs can be deduced. These techniques have been further developed to generate high-throughput approaches that can be used to assess RNA-binding properties of hundreds of proteins [18–22]. Consequently, many of the RNA-binding preferences of human proteins are now known. Functional examination of such proteins has shown that the activity of these proteins directly impact RNA stability, RNA subcellular localisation and the regulation of splicing [18]. Some of these RBPs also regulate virus infection and we will further discuss their role in controlling virus replication. An orthogonal approach for the identification of human proteins that interact with virus RNA involves RNA-capture techniques, such as hybridisation purification of RNA–protein complexes followed by mass spectrometry (HyPR-MS) [23] and comprehensive identification of RNA-binding proteins by mass spectrometry (ChIRP-MS) [24]. In such techniques, the interaction between human proteins and virus RNA is stabilised by a cross-linking step, such as treatment with formaldehyde or UV radiation, followed by incubation with biotinylated oligos targeting virus RNA [25]. RNA:protein complexes are then isolated with streptavidin-conjugated reagents. These approaches have already been applied to multiple viruses including HIV-1 [23,26], SARS-CoV-2 [27,28], Zika virus and ebolavirus [29]. While these approaches have been applied to identify novel host–virus interactions, both ChIRP-MS and HyPR-MS are prone to variability [25]. Nevertheless, at least one protein family seems to be represented across multiple experiments and during infection with different viruses, heterogeneous ribonucleoproteins (hnRNPs). This family includes many RNA-binding proteins and we discuss the activity of several members of this family in the sections below.

**Figure 1:**
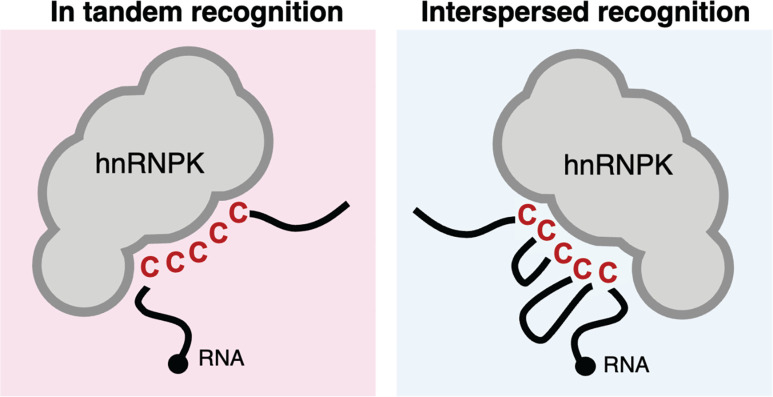
Schematic representation of in tandem and interspersed recognition modes of mononucleotides hnRNPK is a human RNA-binding protein that recognises cytosines in RNA and DNA. While initially thought to recognise sequences of cytosines in tandem (left), its recognition motif is better described if cytosines are interspersed in the RNA molecule (right).

Large scale RBPs mapping experiments have highlighted the distribution of RNA-binding preferences of the human proteome. While the discovery of RNA-binding motifs is inherently difficult and may be dependent on sequence context and RNA structure [19], many human RBPs queried bind to motifs containing stretches of the same nucleotide. For example, the RBPs FUS and EWSR1 preferentially bind G-rich motifs, while hnRNPC and TIA-1 recognise U-rich sequence motifs [18]. To identify preferred motifs bound by RNA-binding proteins, motif discovery algorithms, such as MEME [30], HOMER [31] or GLAM2 [32], are frequently used. These algorithms analyse large sets of RNA sequences and aim to find statistically enriched patterns or conserved motifs that are enriched in the binding sites of RBPs. However, motif discovery algorithms are limited in their ability to detect longer or complex motifs. Algorithms that search for short motifs may miss more intricate binding patterns, which could be relevant for RBPs with specific binding preferences. These algorithms are usually adequate at retrieving motifs containing repeated mononucleotides in tandem; nevertheless, the RNA-binding profile of certain RBPs may be better explained when nucleotides are interspersed. Indeed, there is evidence that the preference for mononucleotide-rich RNA-binding motifs is also observed when these nucleotides are interspaced [19]. An example of that is the protein hnRNPK, whose RNA-binding profile was initially described as the C-rich motif 5′-CCCCCC-3′ but a motif with interspersed cytosine, such as 5′-CNCNCNCNNNCC-3′ (Figure 1), yielded higher enrichment scores [19]. There are also examples of interspersed motif recognition in other antiviral proteins [33]. This indicates that overall sequence composition may be better recognised by some RBPs over linear motifs. However, interspersed patterns have not been extensively studied and further investigation is required to better determine exact binding motifs.

**Figure 2:**
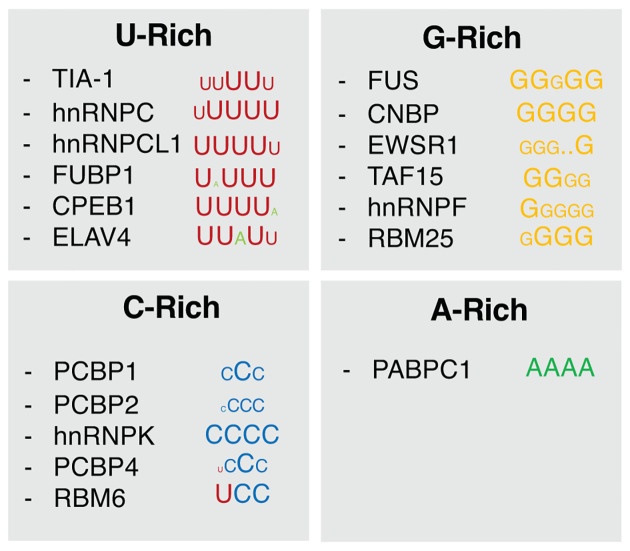
Summary of binding motifs of human RNA-binding proteins Consensus sequence motifs determined by Van Nostrand et al. [18], Dominguez et al. [19] and Ray et al. [20] for human RNA-binding to sequence motifs containing oligomers of mononucleotides. Nucleotide types are represented by colour: uridine (red), guanine (yellow), cytosine (blue) and adenosine (A). Size of the nucleotide symbol (A, C, G, U) indicates the local representation of that type of nucleotide in the consensus motif sequence.

Stretches of the same mononucleotide or overall representation of that base along an RNA molecule may have the propensity to form certain types of RNA structures. G-rich sequences, for instance, have the tendency to form G-quadruplexes which in turn can be detected by RBPs [34]. Therefore, proteins may have evolved to sense nucleotide composition of RNA by scanning RNA molecules for such structures rather than linear motifs. Due to non-Watson–Crick base pairing, G:U and C:U interactions are possible in RNA, and therefore the overall composition of guanine, cytosine and uridine in RNA correlates with more stable secondary structure [35]. Endogenous proteins may have evolved to recognise specific structures formed by these nucleotide pairs. Intriguingly, a feature observed across many large-scale RNA-binding mapping experiments is the scarcity of A-rich motifs (Figure 2) [18–22]. Exceptions to this include proteins involved in recognition of polyA tails, such as PABPC1. It is unlikely that such bias against A-rich motifs is due to technical limitations, since this bias is observed across many types of techniques including several CLIP protocols [22] and SELEX experiments [36]. A possible explanation is the underlying role of structure in the target RNA molecule. A-rich RNA molecules tend to form less stable secondary structures, which may be important for the preservation of RNA:RBP binding site. It is unclear if the scarcity of RNA-binding proteins that recognise A-rich motifs is driven by the lack of such motifs within the coding sequence. PolyA tracks encoding lysine residues have been found to reduce translation and mRNA stability by stalling ribosomes [37], imposing a selective pressure against such motifs within open reading frames. While polyA-binding proteins predominantly bind to 3′ UTRs, it has been proposed that binding to other regions of the mRNA molecule may also occur. PABPC1, for instance, limits its own expression by binding to an A-rich sequence in the 5′ UTR of its mRNA [38]. Since polyA-binding proteins are relevantly abundant in the cell, A-rich motifs present within coding sequences may have been disfavoured and selected against during the evolution of eukaryotic genomes. Nevertheless, definitive investigation regarding the distribution of A-rich sequences within mRNA molecules – including a statistical analysis of their underrepresentation in sequence motifs across multiple CLIP experiments – is missing and requires further examination.

This bias against A-rich motifs may also impact the evolution of RNA viruses. Indeed, a recent study that looked at the overall nucleotide composition of both positive-sense and negative-sense single-stranded RNA viruses showed that the majority of viral coding sequences tend to have high adenosine frequency at the cost of cytosine representation [39]. This bias was not found in coding sequences from bacteriophages or human genomes. The authors of this study postulated that perhaps this bias towards A-richness is a consequence of the type of amino acids encoded by these sequences and, specifically, a bias against amino acids preferably displayed by the major histocompatibility complex (MHC). In line with this hypothesis, analysis of the small peptides that are loaded onto the MHC revealed that these peptides tend to be encoded by A/G-poor coding sequences [39]. While this is a plausible explanation in the context of mammalian immune systems, viruses that infect plants – which are organisms that lack MHC systems – also present similar A-richness [40–42]. Since A-rich motifs are often depleted in CLIP/SELEX experiments of human RBPs [18–20,22], an alternative explanation for the bias towards adenosine in RNA viruses is the mere scarcity of human proteins that recognise such motifs, therefore, reducing the changes of detection and manipulation of virus RNA by the immune system. Nevertheless, this hypothesis has not been tested and requires experimental investigation. Codon choice relies heavily on the third position of the codon, also known as the wobble position. In human cells, codons that contain G or C in the wobble position are associated with mRNAs that are more stably expressed while A/T nucleotides have a negative impact on RNA stability [43]. This distinction may also regulate the expression of functionally distinct genes, for example, genes involved in cell cycle regulation are enriched in codons with A or T in the wobble position [44]. In single-stranded RNA viruses, the third position of the codons tends to be an A or a T, suggesting that viruses may also use this layer of information to regulate gene expression. In composition studies of virus genomes, the adenosine bias is frequently accompanied by the suppression of cytosines [39]. Analysis of SARS-CoV-2 genomes, for example, showed a mutagenic bias to reduce cytosine and increase uridine residues [45,46]. Suppression of cytosine residues have also been observed in other viruses such as HIV-1 [47] and artificially increasing cytosine content in HIV-1 leads to replication defects [33]. The mechanisms underlying variation of mononucleotide composition in virus RNA are not well understood, but we postulate that they may be explained – at least in part – by the nucleotide binding preferences of cellular proteins. In the next few sections, we summarised how human RBPs sensing nucleotide composition affect the replication of many viruses.

## Proteins binding G-rich RNA

**Figure 3:**
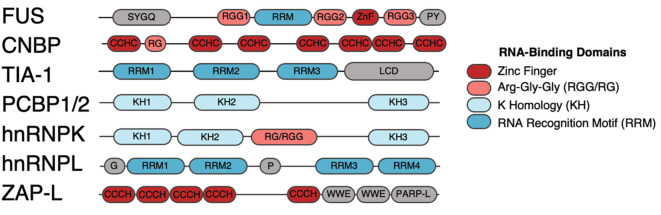
Topological organisation of RNA-binding proteins Schematic representation of the domain organisation landscape of RNA-binding proteins that sense nucleotide composition. Main RNA-binding domains are represented in red (zinc finger domains, which include types CCHC and CCCH), pink (triplet arginine-glycine-glycine (RGG) or arginine-glycine (RG) domains), light blue (K homology (KH) domain) and dark blue (RNA recognition motif (RRM) domain). Domains coloured in grey are domains without known RNA-binding properties.

G-rich motifs seem to be overrepresented in RNA:RBP mapping experiments [18,22,36], and indeed, many proteins that bind G-rich sequences have been previously identified. An example of such protein is the cellular nucleic acid-binding protein (CNBP). CNBP encodes multiple small zinc fingers (also known as zinc knuckles) of the CCHC-type (Figure 3) through which can bind both RNA and DNA [48]. CNBP's binding motif was initially described in sterol regulatory elements found in the promoter regions of the low-density lipoprotein receptor and was thought to consist of 5′-GTG(G/C)GGTG-3′ [49]. Subsequent studies performing in vitro binding assays and yeast one-hybrid screens identified G-rich consensus sequence motifs as preferred DNA binding sites of CNBP [48,50]. Similar experiments identified G-rich motifs as preferred RNA binding sites of CNBP [21]. Using PAR-CLIP, Benhalevy and colleagues found that most targets of CNBP in human cells were mRNA coding sequences containing G-rich motifs, most of which were found to form G-quadruplex (G4) structures [34]. An RNA G4 is a type of secondary RNA structure in which four G residues, either within the same molecule or between multiple RNA molecules, pair with each other forming a square planar structure called guanine tetrad [51]. Such structures can be very stable, due to the multiple hydrogen bonds formed between G residues and base-stacking interactions. The presence of such structures in mRNA has been linked to reduced protein translation, probably due to halted ribosome movement at the G-quadruplex site [52,53]. Previous studies showed that CNBP binds RNA G4 structures *in vitro* and that CNBP promotes the translation of mRNA containing G4 structures in human cells [34]. The current understanding of the activity of CNBP in endogenous RNA molecules points that CNBP binds to G-rich sequences and prevents the formation of G4 structures; however, it may also aid the unwinding of G4 structures [54]. Intriguingly, the coding sequence and structures of the CNBP's zinc fingers closely resemble those of the zinc fingers found in the HIV-1 nucleocapsid. So much so that CNBP's zinc fingers can functionally replace the zinc fingers of HIV-1 nucleocapsid without impacting virus replication [55]. To our knowledge, no association between CNBP and HIV-1 RNA/DNA has been demonstrated. Nevertheless, the similarity in protein topology and functional replacement of the zinc knuckles suggest similar modes of recognition. A study on the recognition of HIV-1 RNA by Gag (a precursor protein of the nucleocapsid) showed preferential binding to A/G-rich regions of the HIV-1 genome [56]. Many G4 structures have been identified in HIV-1 genomes, both in RNA and DNA states [57,58], and indeed – akin to the activity of CNBP - HIV-1 nucleocapsid protein unfolds G4 structures in virus RNA [59]. Crucially, a previous study argued that the A-rich nature of the HIV-1 RNA genome – a constant feature despite high mutation rates – may contribute to the ‘self-recognition’ of virus RNA by viral proteins, which is important for virus genome packaging and virion formation [56]. Further investigation on the effects of CNBP on HIV-1 replication may elucidate novel virus-host interactions.

**Figure 4:**
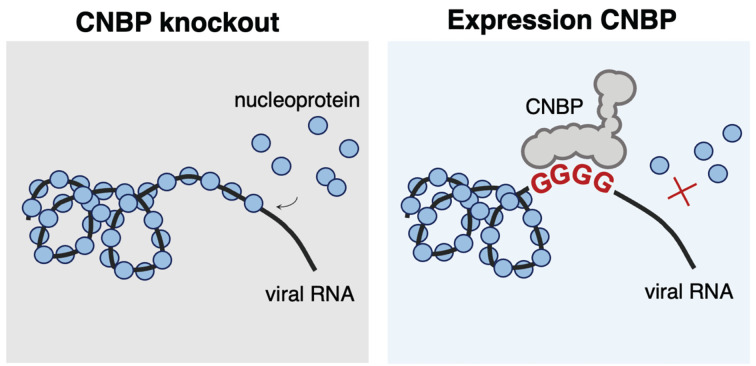
Proposed antiviral mechanism of CNBP In the absence of CNBP, genomic viral RNA (black line) is enveloped by monomers of the viral nucleoprotein (blue circles), forming the ribonucleoprotein complex. This is an essential step in the lifecycle of multiple viruses. However, in the presence of CNBP, G-rich sequences present in the virus RNA are targeted by the CNBP, which competes with the viral nucleoprotein. The interaction of CNBP with virus RNA blocks oligomerisation of nucleoprotein onto virus RNA, preventing virus encapsidation.

Furthermore, a recent study showed that CNBP binds to SARS-CoV-2 RNA [60]. This has been corroborated by another study that demonstrated that CNBP directly interacts with both sense and antisense strands of SARS-CoV-2 RNA genomes [61]. Bezzi and colleagues showed that CNBP-binding sites in SARS-CoV-2 RNAs form G4 structures in vitro, and that CNBP binding to G4 promotes RNA unfolding. Other groups have identified multiple G4 structures in SARS-CoV-2 RNA [62]. Abrogation of CNBP expression in humans cells led to increased SARS-CoV-2 RNA levels following infection [60]. Similarly, depletion of CNBP led to increased virus replication while CNBP overexpression reduced virus replication [63]. CNBP-knockout mice were also more susceptible to infection [63]. However, the mechanism by which CNBP may inhibit virus replication is still poorly understood. While binding of CNBP to G4 structures in human mRNA molecules generally promotes translation, CNBP expression reduced the levels of both virus RNA and virus protein. A recent study proposed that binding of CNBP to virus RNA may prevent encapsidation of viral RNA by the virus nucleoprotein (Figure 4) [63]. Wrapping of virus RNA by the nucleocapsid protein is a common process in the lifecycle of many viruses and it is essential for the packaging of virus genomes into virions. In many viruses, including SARS-CoV-2, this process is now believed to occur through the formation of RNA-protein condensates in the cytoplasm via phase separation [64]. Indeed, expression of CNBP disrupted the formation of liquid-phase condensates between virus RNA and the nucleoprotein [63]. This process seemed to be specific for viral RNA:protein interactions since the addition of CNBP did not affect the formation of condensates when using an unrelated RNA. CNBP may also have indirect effects on virus replication, since this protein can also modulate the innate immune response. The expression of CNBP promoted the production of interleukin-6, a proinflammatory cytokine, in response to LPS [65]. CNBP was shown to become phosphorylated upon influenza virus infection, followed by translocation to the nucleus where it binds to the *IFNB* promoter region [66]. Consequently, IFN-β levels were lower in CNBP-deficient mice. This is in line with previous findings that suggests that CNBP is important for the control of protozoan and bacterial infections [65,67]. Nevertheless, the antiviral mechanism of CNBP is still not fully understood and additional experiments are required to determine how this protein limits virus replication.

Another antiviral protein that recognises G-rich RNA sequences is the Fused in Sarcoma (FUS) protein. FUS expression is up-regulated upon treatment with type I interferon, hinting to a role in controlling viral infections [68]. During coxsackievirus B3 infection, FUS has been shown to directly bind to the virus genome [69]. Consequently, ablation of its expression led to increased virus RNA levels and IRES-mediated virus translation. Similarly, FUS has also been described to inhibit the reactivation of Kaposi’s sarcoma-associated herpesvirus by inhibiting viral gene expression [70]. Nonetheless, it is still unclear how the activity of FUS can lead to decreased virus replication. FUS can bind both DNA and RNA and it has been implicated in various aspects of RNA metabolism, including transcriptional regulation, splicing, RNA transport and translation [71]. It belongs to the FET protein family which also includes EWSR1 and TAF15 proteins that, coincidently, also recognise G-rich motifs [18]. Mutations in the FUS gene have also been linked to several neurodegenerative diseases, including amyotrophic lateral sclerosis and frontotemporal lobar degeneration [72]. In these diseases, FUS protein accumulates abnormally in the cytoplasm of neurons, leading to impaired RNA metabolism and neurodegeneration. The FUS protein comprises several functional domains, including an N-terminal transcriptional activation domain that contains a QGSY-rich region, multiple nucleic-acid-binding domains (Figure 3) such as an RNA-recognition motif (RMM) domain, three Arg-Gly-Gly repeat regions (known as RGG1-3), a zinc-finger motif and a highly conserved C-terminal nonclassical nuclear localisation signal that interacts with the nuclear transport receptor transportin 1 [73]. Based on its domain topology, FUS interaction with its target RNA may be complex since both the RMM domain and the zinc finger can interact with RNA. Attempts to identify RNA-binding sites of FUS were first achieved using SELEX, where the motif 5′-GGUG-3′ was identified [74]. Still, RNAs lacking the GGUG motif retain the ability to interact with FUS [75]. More recently, using multiple CLIP-based approaches, several studies shown that FUS binds to G-rich sequences in RNA with a consensus sequence of 5′-GGGGGG-3′ [18,22]. Structural analysis revealed a bipartite mode of recognition of RNA in which the zinc finger recognises a G-rich motif while the RRM recognises RNA secondary structure [76]. Coupling sequence specificity recognition with structural recognition may explain the diverse impact of FUS in RNA biology and recognition of virus RNA.

## Proteins binding C-rich RNA

Poly-cytosine binding proteins (PCBPs) are RBPs that recognise C-rich nucleic acids. This family of proteins includes PCBP1, PCBP2 and hnRNP-K, and they all have functions in mRNA stability, RNA nuclear traffic and splicing. Some of these proteins are also induced upon interferon stimulation and in response to virus infection, suggesting a potential role in controlling virus replication. PCBP1 and PCBP2 have been implicated in the replication of numerous viruses, and while they share nearly 90% of protein sequence identity, their effects on virus replication are distinct [77]. PCBP2 contains three KH domains, which are domains known to be involved in the recognition of both RNA and DNA. Initially, PCBP2 was thought to bind C/U-rich motifs in RNA [78], while interacting with T-rich DNA molecules *in vitro* [79]. More recently, FAST-iCLIP experiments confirmed that endogenous targets of PCBP2 are C/U-rich [80]. The fate of RNA molecules bound by PCBP1/2 can be diverse: while PCBP1 have been shown to increase the stability of many mRNAs, including α-globin and collagen-α1 [81,82], they can also inhibit translation of other genes [83]. Comparably, the expression of PCBP2 also has opposing effects on virus replication. In human cells, ablation of PCBP2, but not PCBP1, enhanced vesicular stomatitis virus (VSV) replication [84]. Conversely, overexpression of PCBP2 but not PCBP1 was found to reduce VSV replication. This effect is likely due to increased viral RNA stability in the absence of PCBP2 and it does not seem to interfere with viral ribonucleoprotein complexes. PCBP1, in turn, was found to inhibit the replication of HIV-1 [85]. While knockdown of both PCBP1 and PCPB2 increases virus gene expression, only overexpression of PCBP1 reduced viral RNA levels. In particular, the C-terminal KH domain of PCBP1 is responsible for this activity. In contrast, many reports point that the expression of PCBP2 has a positive impact on the replication of many positive-sense single-stranded RNA viruses. Several studies demonstrated that PCBP2 directly binds to the 5′ untranslated regions of poliovirus and hepatitis A and C viruses [86–89]. Attempts to map this interaction showed that PCBP2 binds to C/U-rich regions of the hepatitis C virus (HCV) genome [80]. While the positive effects of the expression of PCBP2 in virus replication may be due to a direct binding to virus RNA, PCBP2 may boost virus replication by down-regulating innate immune responses. Indeed, PCBP2 was shown to negatively regulate innate immune signalling: PCBP2 triggers mitochondrial antiviral signalling protein (MAVS) for degradation and, upon infection with VSV and sendai virus, knockdown of PBCP2 increases MAVS-mediated signalling [90]. PCBP2 is also implicated in the cGAS-STING signalling pathways. Ablation of PCBP2 increases cGAS-STING-mediated signalling after herpes simplex virus 1 (HSV-1) infection, while overexpression of PCBP2 has the opposite effect [91]. PCBP2 interacts with cGAS and prevents its condensation in the cytoplasm, while PCBP1 knockout reduces cGAS-mediated signalling in response to HSV-1 infection. PCBP1 facilitates the binding of DNA to cGAS and directly interacts with HSV-1 and HIV-1 DNA, primarily through its KH1 domain, promoting DNA-induced aggregation of cGAS. PCBP1 and PCBP2 exhibiting both antiviral and proviral activities, and perhaps interactions with different protein partners may explain their divergent roles in regulating immune responses and viral infections [92].

Another member of the poly-cytosine binding protein family is the heterogeneous nuclear ribonucleoprotein K (hnRNPK), which was originally identified as a component of the heterogeneous nuclear ribonucleoprotein complex [93]. This polycytidine-binding protein is involved in a variety of cellular processes, including chromatin remodelling, transcriptional regulation, splicing and RNA translation. It interacts with RNA and DNA and has been shown to act as both a transcriptional activator and a repressor when assembled on DNA [94,95]. hnRNPK has been found to affect mRNA stability and alternative splicing through its direct binding to mRNA [96,97]. The hnRNPK preference towards C-rich RNA sequences has been demonstrated in multiple experiments [18,22] and, as discussed previously, interspersed cytosine residues seem to be preferentially bound by this protein [19]. Consequently, frequency of cytosine present in human RNA molecules correlates with the interaction with hnRNPK in cells [98]. hnRNPK has also been shown to directly interact with virus RNA. hnRNPK binds to HCV RNA and blocks production of infectious particles [99]. Ablation of hnRNPK expression increased HCV replication, and reconstitution was shown to restore suppression of virus infection. In this study, the expression of hnRNPK did not affect the levels of virus RNA. Instead, and akin to the proposed mechanism of action of CNBP, hnRNPK’s antiviral mechanism is thought to be through interference of virus assembly. hnRNPK has also been shown to limit virus replication by targeting viral proteins for proteasomal degradation [100]. Therefore, the antiviral mechanism of hnRNPK may be more complex than previously thought and requires further examination.

hnRNPK has also been shown to directly bind to the HIV-1 RNA genome, where it binds to a C-rich region of a stem-loop structure located in the Env gene region [101]. Immunoprecipitation of HIV-1 RNA led to the identification of hnRNPK and its overexpression altered HIV-1 splicing, which is crucial for efficient HIV-1 replication. The HIV-1 genome is initially transcribed as a large precursor RNA molecule which contains specific splice sites. One essential splice site in HIV-1 pre-mRNA is the major splice donor site located upstream of the viral *env* gene. Alternative splicing at this site results in the production of two main classes of mRNA molecules: partially spliced and fully spliced RNAs. While the unspliced mRNA serves as the genomic RNA that is packaged into virions and contains genetic information for the production of structural proteins (Gag and Pol) and the enzymes required for viral replication (reverse transcriptase, integrase, and protease), spliced RNAs encodes for the envelope and multiple regulatory viral proteins, including Rev and Tat [102]. These proteins play critical roles in regulating viral gene expression and enhancing viral replication. hnRNPK was shown to bind directly to the A7 acceptor site, which is present in all HIV-1-derived RNAs, and strongly inhibit splicing [101]. How hnRNPK inhibits splicing is not yet understood, but it has been suggested that binding of hnRNPK stabilises unspliced RNA molecules. In line with this idea, another group found that hnRNPK is enriched in HIV-1 unspliced RNAs but not in partially or fully spliced RNA molecules [26]. hnRNPK has also been implicated in splicing of other viruses, including influenza A virus (IAV) [103]. At least two IAV segments are subjected to splicing during virus replication: the NS segment, whose unspliced isoform produces the non-structural protein 1 (NS1) while the spliced form encodes for NS2; and the M segment, whose spliced and unspliced forms produce an ion channel (M2) and a matrix protein (M1), respectively [104]. Additional spliced isoforms of M have also been identified [105]. Depletion of hnRNPK led to abnormal mRNA ratios between M1 and M2 RNA and reduced virus replication [103]. hnRNPK was shown to directly bind IAV RNAs in a C-rich region, consistent with the bias for cytosine in endogenous RNAs [106]. Considering that many virus genomes are C-poor, future research into proteins that recognise C-rich RNA sequences may explain composition biases in circulating viruses.

## Proteins binding U-rich RNA

U-rich RNA sequences are crucial for RNA metabolism, primarily through uridylation of mRNA molecules. Uridylation refers to the addition of uridine monophosphate residues to RNA molecules. It is a post-transcriptional modification that mostly occurs at the 3′ end of RNA molecules, however, internal uridylation sites have also been identified in trypanosomes [107,108]. Therefore, multiple proteins have evolved to recognise U-rich RNA. One example of an RBP protein with preference for U-rich RNA sequences is the T-cell-restricted intracellular antigen 1 (TIA-1). Multiple studies using CLIP-based approaches have shown that TIA-1 directly binds to U-rich RNA sequences [18,22,109]. TIA-1 is ubiquitously expressed but it is present at higher levels in immune cells and has been extensively studied for its involvement in cellular stress responses, immune regulation and RNA granule dynamics [110,111]. TIA-1 contains three RRMs, which enable interaction with specific RNA sequences, and a low-complexity C-terminal domain [111]. RRM2 is thought to be the main RNA recognition domain binding to U-rich sequences, while RRM1 does not bind to RNA and RRM3 binds to RNA with no specificity [112]. TIA-1 is known to impact mRNA stability and translation in the cell. Upon binding to target mRNAs, TIA-1 recruits several nucleases and decapping enzymes that metabolise bound RNA [111]. It also participates in the formation of stress granules during cellular stress responses and inhibits the translation of target mRNAs [113,114]. It has been shown that during VSV infection, TIA-1 is enriched in intracellular structures that resemble stress granules [115]. Indeed, knockdown of TIA-1 leads to increase viral gene expression, promoting VSV replication. Similarly, during rabies virus infection, TIA-1 also has an antiviral effect, since cells lacking TIA-1 expression sustain higher replication [116]. This seems to be true for some positive-sense single-stranded viruses as well: during infection with tick-borne encephalitis virus (TBEV), a flavivirus, TIA-1 is recruited to virus replication sites where it directly binds viral RNA [117]. Similarly to VSV and Rabies virus infection, knockdown of TIA-1 led to increased TBEV replication. TIA-1 also binds virus RNA and limits virus replication of other viruses such as enterovirus D68 and red-spotted grouper nervous necrosis virus [118,119]. In contrast, TIA-1 seems to have a positive effect in other viruses, such as enterovirus A71, West Nile virus and dengue virus [120–122]. Virus context specific outcomes may be explained by different localisation of TIA-1 upon infection, and consequently, different interaction partners; however, further investigation is required to assess this hypothesis.

The protein hnRNPL is also known to bind U-rich RNA sequences [18,19]. hnRNP contains four RRMs (termed RRM1-4), an N-terminal glycine-rich region and a flexible, proline-rich domain between RRM2 and 3 [123]. In human cells, hnRNPL inhibits retrotransposition [124] and regulates RNA splicing [125]. hnRNPL inhibits foot-and-mouth disease virus replication by binding to the IRES [126]. This interaction occurs through the domains RRM3 and RRM4. While this interaction does not impact viral protein translation, it leads to reduced viral RNA levels. One possible antiviral mechanism may be the blocking of interaction of viral proteins, such as the virus RNA polymerase, with viral RNA [126].

## Proteins recognising dinucleotide composition

Aside from mononucleotide biases, virus genomes also maintain biased dinucleotide composition. In vertebrate RNA viruses, one of the most pervasive dinucleotide bias is the low frequency of CpG dinucleotides, which resembles the CG-suppressed genome of the human. The paucity of CG dinucleotides in the human genome is thought to be a consequence of the activity of DNA methyltransferases that catalyse the reaction of cytosine to 5-methylcytosine in a CpG context; methylated cytosines are naturally prone to spontaneous deamination, resulting in a C-to-T mutation [127]. While virus RNA is not a substrate for human DNA methyltransferases, CpG suppression in RNA viruses is prevalent and is found to be essential for their replication in human cells; such examples include HIV-1 [128] and influenza virus [129]. A possible explanation for this selection pressure is that viruses have evolved to evade endogenous sensors that detect CpGs in virus RNA. One of the best characterised dinucleotide composition sensor is the zinc-finger antiviral protein (ZAP), which detects CpG dinucleotides and inhibits the replication of a broad range of viruses. ZAP contains four CCCH-type zinc finger motifs (ZnF1-4) at its N-terminus, forming the RNA-binding domain which is crucial for its antiviral activity. The rest of the protein comprises of a WWE domain and a poly(ADP-ribose) polymerase (PARP)-like domain [130,131]. While the PARP-like domain seems to be, at large, dispensable for antiviral activity, the WWE domain may enhance ZAP’s function by binding to poly(ADP-ribose) [132]. There are at least four isoforms of ZAP [133], however the most abundant isoform are the long isoform (ZAP-L) and the short isoform (ZAP-S) whose expression is controlled by an early polyadenylation site [134]. ZAP specifically interacts with single-stranded RNA but it may also interact with structured RNA elements, such as stem loops [135]. ZAP directly binds CpG dinucleotides through its RNA-binding domain, where the highly basic ZnF2 forms a binding pocket specific for accommodating CG dinucleotide bases of the target RNA [128,136,137].

There are two main mechanisms of ZAP that required recruitment of different cofactors: upon interaction with viral RNA, ZAP can inhibit viral protein translation and target viral RNA for degradation [138–141]. To block translation, ZAP interacts with translational initiation factor eIF4A disrupting eIF4A–eIF4G association, which halts translation [142]. TRIM25, a E3 ubiquitin ligase, is also important for translation inhibition by ZAP in the context of alphavirus infection, since ablation of TRIM25 impairs ZAP-dependent repression of viral translation [143]. TRIM25 is comprised of four domains: an N-terminal RING domain which catalyses the transfer of ubiquitin, a B-box domain, a coiled-coil domain and a carboxy-terminal PRY/SPRY domain responsible for substrate binding [144]. TRIM25 interacts with the RNA-binding domain of ZAP potentially through its SPRY domain, and both its RING domain and coiled-coil domain are important for the antiviral activity of ZAP [143,145,146]. Aside from translation inhibition, ZAP also recruits cofactors responsible for RNA degradation; for example, in the context of CG-rich HIV-1 infection, the putative endonuclease KHNYN is recruited by ZAP to cleave viral RNA via its NYN endonuclease domain [147]. ZAP can also recruit helicases, such as the p72 DEAD box RNA helicase, as well as the RNA processing exosome component hRrp46p, that catalyse the degradation of the target RNA [148,149]. Exploiting the antiviral activity of ZAP by introducing CpG dinucleotides in virus genomes has also been shown to be an effective strategy for the generation of live-attenuated vaccines [33,150]. In addition to its antiviral activity, ZAP also regulates endogenous RNAs, for instance, ZAP inhibits the retrotransposition of Long Interspersed Element-1 (LINE-1) via binding to LINE1 RNA and mediate its degradation to prevent its accumulation in the cytoplasm [151,152].

Notably, TRIM25 is an RNA-binding protein itself as well. Previous studies have shown that TRIM25 may bind to RNA through two potential motifs: the 7K sequence in the L2 linker region located between the coiled-coil and the PRY/SPRY domains, and also through residues located within the SPRY domain [153,154]. However, if the RNA-binding activity of TRIM25 is important for the antiviral activity of ZAP is not yet known. Nevertheless, the RNA-binding activity of TRIM25 may be important in a ZAP-independent context; for example, while dengue virus is not sensitive to ZAP [138], its replication is inhibited by TRIM25 in a manner that is dependent on its RNA-binding properties [154]. TRIM25 was also initially implicated in the activation of RIG-I during virus RNA sensing [155]. However, recent evidence suggests that Riplet/RNF135, not TRIM25, is the E3 ubiquitin ligase required for the activation of RIG-I in the presence of double stranded RNA [156,157]. Further investigation is required to determine if TRIM25 senses specific motifs enriched in virus RNA.

## Conclusion

Emerging evidence suggests that some human proteins detect local or global compositional biases in virus RNA. These proteins seem to regulate not only virus replication but also multiple aspect of RNA metabolism. Indeed, mutations found in many of the genes encoding for such proteins contribute to several human diseases [71,125]. These proteins may have evolved to detect stretches of mononucleotides, either in tandem or interspersed [22], in endogenous RNA molecules but later co-opted to control virus infections. Supporting this hypothesis is the observation that many of these proteins are up-regulated upon virus infection or upon the induction of innate immune signalling [91,158,159]. Intriguingly, some of the sensors discussed here were also shown to be negative regulators of the immune response. Antiviral proteins that act on virus RNA have been suggested to affect the composition of co-expressed genes [160], and the evolution of the antiviral state is likely constrained by the activity of many up-regulated RBPs. While this review focuses on RNA viruses, it is likely that such proteins may also impact the replication of DNA viruses by direct interaction with viral DNA and mRNA. A common theme we observed when reviewing the evidence for compositional sensors is that most of the proteins discussed above were found to interact with both RNA and DNA, recognising similar motifs in both molecules. While structural studies that define the molecular details of RNA recognition is missing from many of the proteins reviewed, we hypothesise that such interaction occurs primarily through the recognition of nucleotide residues and not based on interactions with the ribose/deoxyribose bases or diester bond. A mode of recognition like the one described may be suitable for both molecules. In some cases, we discussed how the same protein can be antiviral in some cases and promote virus replication in others. Indeed, endogenous substrates of these RBPs can also have opposite fates in the cell, where proteins can promote mRNA stability of certain targets and RNA degradation of others. While a plausible explanation is the variation in interaction partners, another explanation could be that recognition of the target RNA may be more complex that a single RNA-binding motif. For example, structural studies of FUS interacting with target RNAs suggest a bipartite mode of recognition [76]. Indeed, complex modes of RNA recognition that depart from standard single motif recognition models may explain how different target RNAs can have different fates. To address this problem, the emerging field of ribolinguistics [1,161–163] – which studies how coding biases, synonymous mutations and compositional patterns encoded in RNA sequences impact multiple aspects of RNA biology and virology – will play a pivot role. The further development of RNA:protein mapping approaches, as well as motif discovery algorithms capable of identifying non-linear binding motifs, will paved the way to understanding how complex RNA:protein interactions determine the fate of virus infections.

## Open Access

Open access for this article was enabled by the participation of Imperial College London in an all-inclusive *Read & Publish* agreement with Portland Press and the Biochemical Society under a transformative agreement with JISC.

## Abbreviations

CLIP: cross-linking and immunoprecipitation
HCV: hepatitis C virus
IAV: influenza A virus
NS1: non-structural protein 1
PCBP: poly-cytosine binding protein
RBP: RNA-binding protein
RIG-I: retinoic acid-inducible gene I
RLR: RIG-I-like receptor
RMM: RNA-recognition motif
TLR: Toll-like receptor
VSV: vesicular stomatitis virus

## References

[1] Plotkin J.B. and Kudla G. (2011) Synonymous but not the same: the causes and consequences of codon bias. Nat. Rev. Genet. 12, 32–42 10.1038/nrg2899 21102527 PMC3074964

[2] Simmonds P., Xia W., Baillie J.K. and McKinnon K. (2013) Modelling mutational and selection pressures on dinucleotides in eukaryotic phyla –selection against CpG and UpA in cytoplasmically expressed RNA and in RNA viruses. BMC Genomics 14, 610 10.1186/1471-2164-14-610 24020411 PMC3829696

[3] Breslauer K.J., Frank R., Blöcker H. and Marky L.A. (1986) Predicting DNA duplex stability from the base sequence. Proc. Natl. Acad. Sci. U.S.A. 83, 3746–3750 10.1073/pnas.83.11.3746 3459152 PMC323600

[4] Levy M. and Miller S.L. (1998) The stability of the RNA bases: Implications for the origin of life. Proc. Natl. Acad. Sci. 95, 7933–7938 10.1073/pnas.95.14.7933 9653118 PMC20907

[5] Vinogradov A.E. (2003) DNA helix: the importance of being GC-rich. Nucleic Acids Res. 31, 1838–1844 10.1093/nar/gkg296 12654999 PMC152811

[6] Chan C.Y., Carmack C.S., Long D.D., Maliyekkel A., Shao Y., Roninson I.B. et al. (2009) A structural interpretation of the effect of GC-content on efficiency of RNA interference. BMC Bioinform. 10, S33 10.1186/1471-2105-10-S1-S33 19208134 PMC2648742

[7] Poetsch A.R., Boulton S.J. and Luscombe N.M. (2018) Genomic landscape of oxidative DNA damage and repair reveals regioselective protection from mutagenesis. Genome Biol. 19, 215 10.1186/s13059-018-1582-2 30526646 PMC6284305

[8] Matallana-Surget S., Meador J.A., Joux F. and Douki T. (2008) Effect of the GC content of DNA on the distribution of UVB-induced bipyrimidine photoproducts. Photochem. Photobiol. Sci. 7, 794–801 10.1039/b719929e 18597027

[9] Saito T., Owen D.M., Jiang F., Marcotrigiano J. and Gale M. Jr (2008) Innate immunity induced by composition-dependent RIG-I recognition of hepatitis C virus RNA. Nature 454, 523–527 10.1038/nature07106 18548002 PMC2856441

[10] Zhang Z., Ohto U., Shibata T., Taoka M., Yamauchi Y., Sato R. et al. (2018) Structural analyses of toll-like receptor 7 reveal detailed RNA sequence specificity and recognition mechanism of agonistic ligands. Cell Rep. 25, 3371.e5–3381.e5 10.1016/j.celrep.2018.11.081 30566863

[11] Kawai T. and Akira S. (2009) The roles of TLRs, RLRs and NLRs in pathogen recognition. Int. Immunol. 21, 317–337 10.1093/intimm/dxp017 19246554 PMC2721684

[12] Ule J., Jensen K.B., Ruggiu M., Mele A., Ule A. and Darnell R.B. (2003) CLIP identifies Nova-regulated RNA networks in the brain. Science 302, 1212–1215 10.1126/science.1090095 14615540

[13] Hafner M., Landthaler M., Burger L., Khorshid M., Hausser J., Berninger P. et al. (2010) Transcriptome-wide Identification of RNA-binding protein and MicroRNA Target Sites by PAR-CLIP. Cell 141, 129–141 10.1016/j.cell.2010.03.009 20371350 PMC2861495

[14] Licatalosi D.D., Mele A., Fak J.J., Ule J., Kayikci M., Chi S.W. et al. (2008) HITS-CLIP yields genome-wide insights into brain alternative RNA processing. Nature 456, 464–469 10.1038/nature07488 18978773 PMC2597294

[15] König J., Zarnack K., Rot G., Curk T., Kayikci M., Zupan B. et al. (2010) iCLIP reveals the function of hnRNP particles in splicing at individual nucleotide resolution. Nat. Struct. Mol. Biol. 17, 909–915 10.1038/nsmb.1838 20601959 PMC3000544

[16] Tuerk C. and Gold L. (1990) Systematic evolution of ligands by exponential enrichment: RNA ligands to bacteriophage T4 DNA polymerase. Science 249, 505–510 10.1126/science.2200121 2200121

[17] Ellington A.D. and Szostak J.W. (1990) In vitro selection of RNA molecules that bind specific ligands. Nature 346, 818–822 10.1038/346818a0 1697402

[18] van Nostrand E.L., Freese P., Pratt G.A., Wang X., Wei X. et al. (2020) A large-scale binding and functional map of human RNA-binding proteins. Nature 583, 711–719 10.1038/s41586-020-2077-3 32728246 PMC7410833

[19] Dominguez D., Freese P., Alexis M.S., Su A., Hochman M., Palden T. et al. (2018) Sequence, structure, and context preferences of human RNA binding proteins. Mol. Cell 70, 854.e9–867.e9 10.1016/j.molcel.2018.05.001 29883606 PMC6062212

[20] Ray D., Laverty K.U., Jolma A., Nie K., Samson R., Pour S.E. et al. (2023) RNA-binding proteins that lack canonical RNA-binding domains are rarely sequence-specific. Sci. Rep. 13, 5238 10.1038/s41598-023-32245-9 37002329 PMC10066285

[21] Ray D., Ha K.C.H., Nie K., Zheng H., Hughes T.R. and Morris Q.D. (2017) RNAcompete methodology and application to determine sequence preferences of unconventional RNA-binding proteins. Methods 118-119, 3–15 10.1016/j.ymeth.2016.12.003 27956239 PMC5411283

[22] Kuret K., Amalietti A.G., Jones D.M., Capitanchik C. and Ule J. (2022) Positional motif analysis reveals the extent of specificity of protein-RNA interactions observed by CLIP. Genome Biol. 23, 191 10.1186/s13059-022-02755-2 36085079 PMC9461102

[23] Knoener R.A., Becker J.T., Scalf M., Sherer N.M. and Smith L.M. (2017) Elucidating the in vivo interactome of HIV-1 RNA by hybridization capture and mass spectrometry. Sci. Rep. 7, 16965 10.1038/s41598-017-16793-5 29208937 PMC5717263

[24] Chu C., Zhang Q.C., da Rocha S.T., Flynn R.A., Bharadwaj M., Calabrese J.M. et al. (2015) Systematic discovery of Xist RNA binding proteins. Cell 161, 404–416 10.1016/j.cell.2015.03.025 25843628 PMC4425988

[25] Iselin L., Palmalux N., Kamel W., Simmonds P., Mohammed S. and Castello A. (2022) Uncovering viral RNA-host cell interactions on a proteome-wide scale. Trends Biochem. Sci 47, 23–38 10.1016/j.tibs.2021.08.002 34509361 PMC9187521

[26] Knoener R., Evans E. III, Becker J.T., Scalf M., Benner B. et al. (2021) Identification of host proteins differentially associated with HIV-1 RNA splice variants. eLife 10, e62470 10.7554/eLife.62470 33629952 PMC7906601

[27] Flynn R.A., Belk J.A., Qi Y., Yasumoto Y., Wei J., Alfajaro M.M. et al. (2021) Discovery and functional interrogation of SARS-CoV-2 RNA-host protein interactions. Cell 184, 2394.e16–2411.e16 10.1016/j.cell.2021.03.012 33743211 PMC7951565

[28] Labeau A., Fery-Simonian L., Lefevre-Utile A., Pourcelot M., Bonnet-Madin L., Soumelis V. et al. (2022) Characterization and functional interrogation of the SARS-CoV-2 RNA interactome. Cell Rep. 39, 110744 10.1016/j.celrep.2022.110744 35477000 PMC9040432

[29] Zhang S., Huang W., Ren L., Ju X., Gong M., Rao J. et al. (2022) Comparison of viral RNA–host protein interactomes across pathogenic RNA viruses informs rapid antiviral drug discovery for SARS-CoV-2. Cell Res. 32, 9–23 10.1038/s41422-021-00581-y 34737357 PMC8566969

[30] Bailey T.L. and Elkan C. (1994) Fitting a mixture model by expectation maximization to discover motifs in biopolymers. Proc. Int. Conf. Intell. Syst. Mol. Biol. 2, 28–36 7584402

[31] Heinz S., Benner C., Spann N., Bertolino E., Lin Y.C., Laslo P. et al. (2010) Simple combinations of lineage-determining transcription factors prime cis-regulatory elements required for macrophage and B cell identities. Mol. Cell 38, 576–589 10.1016/j.molcel.2010.05.004 20513432 PMC2898526

[32] Frith M.C., Saunders N.F.W., Kobe B. and Bailey T.L. (2008) Discovering sequence motifs with arbitrary insertions and deletions. PLoS Comput. Biol. 4, e1000071 10.1371/journal.pcbi.1000071 18437229 PMC2323616

[33] Gonçalves-Carneiro D., Mastrocola E., Lei X., DaSilva J., Chan Y.F. and Bieniasz P.D. (2022) Rational attenuation of RNA viruses with zinc finger antiviral protein. Nat. Microbiol. 7, 1558–1567 10.1038/s41564-022-01223-8 36075961 PMC9519448

[34] Benhalevy D., Gupta S.K., Danan C.H., Ghosal S., Sun H.W., Kazemier H.G. et al. (2017) The human CCHC-type zinc finger nucleic acid-binding protein binds G-rich elements in target mRNA coding sequences and promotes translation. Cell Rep. 18, 2979–2990 10.1016/j.celrep.2017.02.080 28329689 PMC5393907

[35] Leontis N.B., Stombaugh J. and Westhof E. (2002) The non-Watson-Crick base pairs and their associated isostericity matrices. Nucleic Acids Res. 30, 3497–3531 10.1093/nar/gkf481 12177293 PMC134247

[36] Jolma A., Zhang J., Mondragón E., Morgunova E., Kivioja T., Laverty K.U. et al. (2020) Binding specificities of human RNA-binding proteins toward structured and linear RNA sequences. Genome Res. 30, 962–973 10.1101/gr.258848.119 32703884 PMC7397871

[37] Arthur L.L., Pavlovic-Djuranovic S., Koutmou K.S., Green R., Szczesny P. and Djuranovic S. (2015) Translational control by lysine-encoding A-rich sequences. Sci. Adv. 1, e1500154 10.1126/sciadv.1500154 26322332 PMC4552401

[38] Kini H.K., Silverman I.M., Ji X., Gregory B.D. and Liebhaber S.A. (2016) Cytoplasmic poly(A) binding protein-1 binds to genomically encoded sequences within mammalian mRNAs. RNA 22, 61–74 10.1261/rna.053447.115 26554031 PMC4691835

[39] Kustin T. and Stern A. (2020) Biased mutation and selection in RNA viruses. Mol. Biol. Evol. 38, 575–588 10.1093/molbev/msaa247 PMC7543401 32986832

[40] He Z., Qin L., Xu X. and Ding S. (2022) Evolution and host adaptability of plant RNA viruses: Research insights on compositional biases. Computational Struct. Biotechnol. J. 20, 2600–2610 10.1016/j.csbj.2022.05.021 PMC9160401 35685354

[41] Prádena A.G.d., Jimenez A.S., León D.S., Simmonds P., García J.A. and Valli A.A. (2020) Plant virus genome is shaped by specific dinucleotide restrictions that influence viral infection. mBio 11, e02818–e02819 32071264 10.1128/mBio.02818-19 PMC7029135

[42] Qin L., Ding S. and He Z. (2023) Compositional biases and evolution of the largest plant RNA virus order Patatavirales. Int. J. Biol. Macromol. 240, 124403 10.1016/j.ijbiomac.2023.124403 37076075

[43] Hia F., Yang S.F., Shichino Y., Yoshinaga M., Murakawa Y., Vandenbon A. et al. (2019) Codon bias confers stability to human mRNAs. EMBO Rep. 20, e48220 10.15252/embr.201948220 31482640 PMC6831995

[44] Gingold H., Tehler D., Christoffersen N.R., Nielsen M.M., Asmar F., Kooistra S.M. et al. (2014) A dual program for translation regulation in cellular proliferation and differentiation. Cell 158, 1281–1292 10.1016/j.cell.2014.08.011 25215487

[45] Wang Y., Chen X.-Y., Yang L., Yao Q. and Chen K.P. (2022) Human SARS-CoV-2 has evolved to increase U content and reduce genome size. Int. J. Biol. Macromol. 204, 356–363 10.1016/j.ijbiomac.2022.02.034 35149094 PMC8824384

[46] Danchin A. and Marlière P. (2020) Cytosine drives evolution of SARS-CoV-2. Environ. Microbiol. 22, 1977–1985 10.1111/1462-2920.15025 32291894 PMC7262064

[47] van der Kuyl A.C. and Berkhout B. (2012) The biased nucleotide composition of the HIV genome: a constant factor in a highly variable virus. Retrovirology 9, 92 10.1186/1742-4690-9-92 23131071 PMC3511177

[48] Armas P., Nasif S. and Calcaterra N.B. (2008) Cellular nucleic acid binding protein binds G-rich single-stranded nucleic acids and may function as a nucleic acid chaperone. J. Cell. Biochem. 103, 1013–1036 10.1002/jcb.21474 17661353

[49] Rajavashisth T.B., Taylor A.K., Andalibi A., Svenson K.L. and Lusis A.J. (1989) Identification of a zinc finger protein that binds to the sterol regulatory element. Science 245, 640–643 10.1126/science.2562787 2562787

[50] Armas P., Margarit E., Mouguelar V.S., Allende M.L. and Calcaterra N.B. (2013) Beyond the binding site: in vivo identification of tbx2, smarca5 and wnt5b as molecular targets of CNBP during embryonic development. PLoS ONE 8, e63234 10.1371/journal.pone.0063234 23667590 PMC3646763

[51] Guo J.U. and Bartel D.P. (2016) RNA G-quadruplexes are globally unfolded in eukaryotic cells and depleted in bacteria. Science 353, aaf5371–1 until aaf5371-8 10.1126/science.aaf5371 27708011 PMC5367264

[52] Varshney D., Cuesta S.M., Herdy B., Abdullah U.B., Tannahill D. and Balasubramanian S. (2021) RNA G-quadruplex structures control ribosomal protein production. Sci. Rep. 11, 22735 10.1038/s41598-021-01847-6 34815422 PMC8611094

[53] Murat P., Marsico G., Herdy B., Ghanbarian A., Portella G. and Balasubramanian S. (2018) RNA G-quadruplexes at upstream open reading frames cause DHX36- and DHX9-dependent translation of human mRNAs. Genome Biol. 19, 229 10.1186/s13059-018-1602-2 30591072 PMC6307142

[54] David A.P., Pipier A., Pascutti F., Binolfi A., Weiner A.M.J., Challier E. et al. (2019) CNBP controls transcription by unfolding DNA G-quadruplex structures. Nucleic Acids Res. 47, 7901–7913 10.1093/nar/gkz527 31219592 PMC6735679

[55] McGrath C.F., Buckman J.S., Gagliardi T.D., Bosche W.J., Coren L.V. and Gorelick R.J. (2003) Human cellular nucleic acid-binding protein Zn^2+^ fingers support replication of human immunodeficiency virus type 1 when they are substituted in the nucleocapsid protein. J. Virol. 77, 8524–8531 10.1128/JVI.77.15.8524-8531.2003 12857921 PMC165261

[56] kutluay s.b., zang t., blanco-melo d., powell c., jannain d. et al. (2014) Global changes in the RNA binding specificity of HIV-1 gag regulate virion genesis. Cell 159, 1096–1109 10.1016/j.cell.2014.09.057 25416948 PMC4247003

[57] Piekna-Przybylska D., Sullivan M.A., Sharma G. and Bambara R.A. (2014) U3 region in the HIV-1 genome adopts a G-quadruplex structure in its RNA and DNA sequence. Biochemistry 53, 2581–2593 10.1021/bi4016692 24735378 PMC4007979

[58] Harpster C., Boyle E., Musier-Forsyth K. and Kankia B. (2021) HIV-1 genomic RNA U3 region forms a stable quadruplex-hairpin structure. Biophys. Chem. 272, 106567 10.1016/j.bpc.2021.106567 33713997 PMC8051326

[59] Butovskaya E., Soldà P., Scalabrin M., Nadai M. and Richter S.N. (2019) HIV-1 nucleocapsid protein unfolds stable RNA G-quadruplexes in the viral genome and is inhibited by G-Quadruplex Ligands. ACS Infect Dis. 5, 2127–2135 10.1021/acsinfecdis.9b00272 31646863 PMC6909241

[60] Schmidt N., Lareau C.A., Keshishian H., Ganskih S., Schneider C., Hennig T. et al. (2021) The SARS-CoV-2 RNA–protein interactome in infected human cells. Nat. Microbiol. 6, 339–353 10.1038/s41564-020-00846-z 33349665 PMC7906908

[61] Bezzi G., Piga E.J., Binolfi A. and Armas P. (2021) CNBP Binds and unfolds in vitro G-quadruplexes formed in the SARS-CoV-2 positive and negative genome strands. Int. J. Mol. Sci. 22, 1–22 10.3390/ijms22052614 33807682 PMC7961906

[62] Qin G., Zhao C., Liu Y., Zhang C., Yang G., Yang J. et al. (2022) RNA G-quadruplex formed in SARS-CoV-2 used for COVID-19 treatment in animal models. Cell Discovery 8, 86 10.1038/s41421-022-00450-x 36068208 PMC9447362

[63] Fitzgerald K., Chen Y., Lei X., Jiang Z., Humphries F., Mustone N. et al. (2022) CNBP restricts SARS-CoV2 by regulating IFN and disrupting RNA-protein condensates. Res. Sq. rs.3.rs–1576788 10.21203/rs.3.rs-1576788/v1 Preprint

[64] Li H., Ernst C., Kolonko-Adamska M., Greb-Markiewicz B., Man J., Parissi V. et al. (2022) Phase separation in viral infections. Trends Microbiol. 30, 1217–1231 10.1016/j.tim.2022.06.005 35902318

[65] Lee E., Lee T.A., Kim J.H., Park A., Ra E.A., Kang S. et al. (2017) CNBP acts as a key transcriptional regulator of sustained expression of interleukin-6. Nucleic Acids Res. 45, 3280–3296 10.1093/nar/gkx071 28168305 PMC5389554

[66] Chen Y., Lei X., Jiang Z. and Fitzgerald K.A. (2021) Cellular nucleic acid-binding protein is essential for type I interferon-mediated immunity to RNA virus infection. Proc. Natl. Acad. Sci. U.S.A. 118 10.1073/pnas.2100383118 PMC8255963 34168080

[67] Chen Y., Sharma S., Assis P.A., Jiang Z., Elling R., Olive A.J. et al. (2018) CNBP controls IL-12 gene transcription and Th1 immunity. J. Exp. Med. 215, 3136–3150 10.1084/jem.20181031 30442645 PMC6279399

[68] Shelkovnikova T.A., An H., Skelt L., Tregoning J.S., Humphreys I.R. and Buchman V.L. (2019) Antiviral immune response as a trigger of FUS proteinopathy in amyotrophic lateral sclerosis. Cell Rep. 29, 4496.e4–4508.e4 10.1016/j.celrep.2019.11.094 31875556 PMC6941233

[69] Xue Y.C., Ng C.S., Mohamud Y., Fung G., Liu H., Bahreyni A. et al. (2021) FUS/TLS suppresses enterovirus replication and promotes antiviral innate immune responses. J. Virol. 95, e00304–e00321 10.1128/JVI.00304-21 33827951 PMC8316056

[70] Dunker W., Song Y., Zhao Y. and Karijolich J. (2018) FUS negatively regulates Kaposi's sarcoma-associated herpesvirus gene expression. Viruses 10, 337–348 10.3390/v10070359 29986386 PMC6070805

[71] Deng H., Gao K. and Jankovic J. (2014) The role of FUS gene variants in neurodegenerative diseases. Nat. Rev. Neurol. 10, 337–348 10.1038/nrneurol.2014.78 24840975

[72] Lagier-Tourenne C., Polymenidou M. and Cleveland D.W. (2010) TDP-43 and FUS/TLS: emerging roles in RNA processing and neurodegeneration. Hum. Mol. Genet. 19, R46–R64 10.1093/hmg/ddq137 20400460 PMC3167692

[73] Iko Y., Kodama T.S., Kasai N., Oyama T., Morita E.H., Muto T. et al. (2004) Domain architectures and characterization of an RNA-binding protein, TLS. J. Biol. Chem. 279, 44834–44840 10.1074/jbc.M408552200 15299008

[74] Lerga A., Hallier M., Delva L., Orvain C., Gallais I., Marie J. et al. (2001) Identification of an RNA binding specificity for the potential splicing factor TLS. J. Biol. Chem. 276, 6807–6816 10.1074/jbc.M008304200 11098054

[75] Hoell J.I., Larsson E., Runge S., Nusbaum J.D., Duggimpudi S., Farazi T.A. et al. (2011) RNA targets of wild-type and mutant FET family proteins. Nat. Struct. Mol. Biol. 18, 1428–1431 10.1038/nsmb.2163 22081015 PMC3230689

[76] Loughlin F.E., Lukavsky P.J., Kazeeva T., Reber S., Hock E.M., Colombo M. et al. (2019) The solution structure of FUS bound to RNA reveals a bipartite mode of RNA recognition with both sequence and shape specificity. Mol. Cell 73, 490.e6–504.e6 10.1016/j.molcel.2018.11.012 30581145

[77] Chkheidze A.N. and Liebhaber S.A. (2003) A novel set of nuclear localization signals determine distributions of the alphaCP RNA-binding proteins. Mol. Cell. Biol. 23, 8405–8415 10.1128/MCB.23.23.8405-8415.2003 14612387 PMC262676

[78] Choi H.S., Hwang C.K., Song K.Y., Law P.Y., Wei L.N. and Loh H.H. (2009) Poly(C)-binding proteins as transcriptional regulators of gene expression. Biochem. Biophys. Res. Commun. 380, 431–436 10.1016/j.bbrc.2009.01.136 19284986 PMC2657093

[79] Du Z., Lee J.K., Tjhen R., Li S., Pan H., Stroud R.M. et al. (2005) Crystal Structure of the First KH Domain of Human Poly(C)-binding Protein-2 in Complex with a C-rich Strand of Human Telomeric DNA at 1.7 Å*. J. Biol. Chem. 280, 38823–38830 10.1074/jbc.M508183200 16186123

[80] Flynn R.A., Martin L., Spitale R.C., Do B.T., Sagan S.M., Zarnegar B. et al. (2015) Dissecting noncoding and pathogen RNA-protein interactomes. RNA 21, 135–143 10.1261/rna.047803.114 25411354 PMC4274633

[81] Chkheidze A.N., Lyakhov D.L., Makeyev A.V., Morales J., Kong J. and Liebhaber S.A. (1999) Assembly of the α-globin mRNA stability complex reflects binary interaction between the pyrimidine-rich 3′ untranslated region determinant and poly(C) binding protein αCP. Mol. Cell. Biol. 19, 4572–4581 10.1128/MCB.19.7.4572 10373506 PMC84255

[82] Lindquist J.N., Kauschke S.G., Stefanovic B., Burchardt E.R. and Brenner D.A. (2000) Characterization of the interaction between αCP2 and the 3′-untranslated region of collagen α1(I) mRNA. Nucleic Acids Res. 28, 4306–4316 10.1093/nar/28.21.4306 11058131 PMC113122

[83] Ostareck-Lederer A., Ostareck D.H., Standart N. and Thiele B.J. (1994) Translation of 15-lipoxygenase mRNA is inhibited by a protein that binds to a repeated sequence in the 3′ untranslated region. EMBO J. 13, 1476–1481 10.1002/j.1460-2075.1994.tb06402.x 8137829 PMC394967

[84] Dinh P.X., Beura L.K., Panda D., Das A. and Pattnaik A.K. (2011) Antagonistic effects of cellular poly(C) binding proteins on vesicular stomatitis virus gene expression. J. Virol. 85, 9459–9471 10.1128/JVI.05179-11 21752917 PMC3165775

[85] Woolaway K., Asai K., Emili A. and Cochrane A. (2007) hnRNP E1 and E2 have distinct roles in modulating HIV-1 gene expression. Retrovirology 4, 28 10.1186/1742-4690-4-28 17451601 PMC1863430

[86] Gamarnik A.V. and Andino R. (1997) Two functional complexes formed by KH domain containing proteins with the 5′ noncoding region of poliovirus RNA. RNA 3, 882–892 9257647 PMC1369533

[87] Blyn L.B., Towner J.S., Semler B.L. and Ehrenfeld E. (1997) Requirement of Poly(rC) binding protein 2 for translation of poliovirus RNA. J. Virol. 71, 6243–6246 10.1128/jvi.71.8.6243-6246.1997 9223526 PMC191892

[88] Graff J., Cha J., Blyn L.B. and Ehrenfeld E. (1998) Interaction of poly(rC) binding protein 2 with the 5′ noncoding region of hepatitis A virus RNA and its effects on translation. J. Virol. 72, 9668–9675 10.1128/JVI.72.12.9668-9675.1998 9811700 PMC110476

[89] Wang L., Jeng K.S. and Lai M.M. (2011) Poly(C)-binding protein 2 interacts with sequences required for viral replication in the hepatitis C virus (HCV) 5′ untranslated region and directs HCV RNA replication through circularizing the viral genome. J. Virol. 85, 7954–7964 10.1128/JVI.00339-11 21632751 PMC3147998

[90] You F., Sun H., Zhou X., Sun W., Liang S., Zhai Z. et al. (2009) PCBP2 mediates degradation of the adaptor MAVS via the HECT ubiquitin ligase AIP4. Nat. Immunol. 10, 1300–1308 10.1038/ni.1815 19881509

[91] Gu H., Yang J., Zhang J., Song Y., Zhang Y., Xu P. et al. (2022) PCBP2 maintains antiviral signaling homeostasis by regulating cGAS enzymatic activity via antagonizing its condensation. Nat. Commun. 13, 1564 10.1038/s41467-022-29266-9 35322803 PMC8943206

[92] Schweppe D.K., Huttlin E.L., Harper J.W. and Gygi S.P. (2018) BioPlex display: an interactive suite for large-scale AP–MS protein–protein interaction Data. J. Proteome Res. 17, 722–726 10.1021/acs.jproteome.7b00572 29054129 PMC7029486

[93] Dreyfuss G., Matunis M.J., Pinol-Roma S. and Burd C.G. (1993) hnRNP PROTEINS AND THE BIOGENESIS OF mRNA. Annu. Rev. Biochem. 62, 289–321 10.1146/annurev.bi.62.070193.001445 8352591

[94] Michelotti E.F., Michelotti G.A., Aronsohn A.I. and Levens D. (1996) Heterogeneous nuclear ribonucleoprotein K is a transcription factor. Mol. Cell. Biol. 16, 2350–2360 10.1128/MCB.16.5.2350 8628302 PMC231223

[95] Du Q., Melnikova I.N. and Gardner P.D. (1998) Differential effects of heterogeneous nuclear ribonucleoprotein K on Sp1- and Sp3-mediated transcriptional activation of a neuronal nicotinic acetylcholine receptor promoter. J. Biol. Chem. 273, 19877–19883 10.1074/jbc.273.31.19877 9677424

[96] Skalweit A., Doller A., Huth A., Kähne T., Persson P.B. and Thiele B.J. (2003) Posttranscriptional control of renin synthesis: identification of proteins interacting with renin mRNA 3′-untranslated region. Circ. Res. 92, 419–427 10.1161/01.RES.0000059300.67152.4E 12600897

[97] Expert-Bezançon A., Le Caer J.P. and Marie J. (2002) Heterogeneous nuclear ribonucleoprotein (hnRNP) K is a component of an intronic splicing enhancer complex that activates the splicing of the alternative exon 6A from chicken beta-tropomyosin pre-mRNA. J. Biol. Chem. 277, 16614–16623 10.1074/jbc.M201083200 11867641

[98] Nakamoto M.Y., Lammer N.C., Batey R.T. and Wuttke D.S. (2020) hnRNPK recognition of the B motif of Xist and other biological RNAs. Nucleic Acids Res. 48, 9320–9335 10.1093/nar/gkaa677 32813011 PMC7498318

[99] Poenisch M., Metz P., Blankenburg H., Ruggieri A., Lee J.-Y., Rupp D. et al. (2015) Identification of HNRNPK as Regulator of Hepatitis C Virus Particle Production. PLoS Pathog. 11, e1004573 10.1371/journal.ppat.1004573 25569684 PMC4287573

[100] Qin W., Kong N., Wang C., Dong S., Zhai H., Zhai X. et al. (2022) hnRNP K degrades viral nucleocapsid protein and induces type I IFN production to inhibit porcine epidemic diarrhea virus replication. J. Virol. 96, e0155522 10.1128/jvi.01555-22 36317879 PMC9682996

[101] Marchand V., Santerre M., Aigueperse C., Fouillen L., Saliou J.M., Van Dorsselaer A. et al. (2011) Identification of protein partners of the human immunodeficiency virus 1 tat/rev exon 3 leads to the discovery of a new HIV-1 splicing regulator, protein hnRNP K. RNA Biol. 8, 325–342 10.4161/rna.8.2.13984 21368586

[102] Emery A. and Swanstrom R. (2021) HIV-1: to splice or not to splice, that is the question. Viruses 13, 181 10.3390/v13020181 33530363 PMC7912102

[103] Tsai P.L., Chiou N.T., Kuss S., García-Sastre A., Lynch K.W. and Fontoura B.M. (2013) Cellular RNA binding proteins NS1-BP and hnRNP K regulate influenza A virus RNA splicing. PLoS Pathog. 9, e1003460 10.1371/journal.ppat.1003460 23825951 PMC3694860

[104] Dou D., Revol R., Östbye H., Wang H. and Daniels R. (2018) Influenza A virus cell entry, replication, virion assembly and movement. Front. Immunol. 9, 1–17 10.3389/fimmu.2018.01581 article 1581 30079062 PMC6062596

[105] Wise H.M., Hutchinson E.C., Jagger B.W., Stuart A.D., Kang Z.H., Robb N. et al. (2012) Identification of a novel splice variant form of the influenza A virus M2 ion channel with an antigenically distinct ectodomain. PLoS Pathog. 8, e1002998 10.1371/journal.ppat.1002998 23133386 PMC3486900

[106] Thompson M.G., Muñoz-Moreno R., Bhat P., Roytenberg R., Lindberg J., Gazzara M.R. et al. (2018) Co-regulatory activity of hnRNP K and NS1-BP in influenza and human mRNA splicing. Nat. Commun. 9, 2407 10.1038/s41467-018-04779-4 29921878 PMC6008300

[107] Ernst N.L., Panicucci B., Igo R.P. Jr, Panigrahi A.K., Salavati R. and Stuart K. (2003) TbMP57 Is a 3′ Terminal Uridylyl Transferase (TUTase) of the *Trypanosoma brucei* Editosome. Mol. Cell 11, 1525–1536 10.1016/S1097-2765(03)00185-0 12820966

[108] Schnaufer A., Ernst N.L., Palazzo S.S., O'Rear J., Salavati R. and Stuart K. (2003) Separate insertion and deletion subcomplexes of the Trypanosoma brucei RNA editing complex. Mol. Cell 12, 307–319 10.1016/S1097-2765(03)00286-7 14536071

[109] Wang Z., Kayikci M., Briese M., Zarnack K., Luscombe N.M., Rot G. et al. (2010) iCLIP predicts the dual splicing effects of TIA-RNA interactions. PLoS Biol. 8, e1000530 21048981 10.1371/journal.pbio.1000530 PMC2964331

[110] Felgar R.E., Macon W.R., Kinney M.C., Roberts S., Pasha T. and Salhany K.E. (1997) TIA-1 expression in lymphoid neoplasms. Identification of subsets with cytotoxic T lymphocyte or natural killer cell differentiation. Am. J. Pathol. 150, 1893–1900 9176382 PMC1858307

[111] Fernández-Gómez A. and Izquierdo J.M. (2022) The multifunctional faces of T-cell intracellular antigen 1 in health and disease. Int. J. Mol. Sci. 23, 1–25 10.3390/ijms23031400 PMC8836218 35163320

[112] Dember L.M., Kim N.D., Liu K.-Q. and Anderson P. (1996) Individual RNA recognition motifs of TIA-1 and TIAR have different RNA Binding Specificities (*). J. Biol. Chem. 271, 2783–2788 10.1074/jbc.271.5.2783 8576255

[113] Piecyk M., Wax S., Beck A.R., Kedersha N., Gupta M., Maritim B. et al. (2000) TIA-1 is a translational silencer that selectively regulates the expression of TNF-alpha. EMBO J. 19, 4154–4163 10.1093/emboj/19.15.4154 10921895 PMC306595

[114] Gilks N., Kedersha N., Ayodele M., Shen L., Stoecklin G., Dember L.M. et al. (2004) Stress granule assembly is mediated by prion-like aggregation of TIA-1. Mol. Biol. Cell 15, 5383–5398 10.1091/mbc.e04-08-0715 15371533 PMC532018

[115] Dinh P.X., Beura L.K., Das P.B., Panda D., Das A. and Pattnaik A.K. (2013) Induction of stress granule-like structures in vesicular stomatitis virus-infected cells. J. Virol. 87, 372–383 10.1128/JVI.02305-12 23077311 PMC3536414

[116] Nikolic J., Civas A., Lama Z., Lagaudrière-Gesbert C. and Blondel D. (2016) Rabies virus infection induces the formation of stress granules closely connected to the viral factories. PLoS Pathog. 12, e1005942 10.1371/journal.ppat.1005942 27749929 PMC5066959

[117] Albornoz A., Carletti T., Corazza G. and Marcello A. (2014) The stress granule component TIA-1 binds tick-borne encephalitis virus RNA and is recruited to perinuclear sites of viral replication to inhibit viral translation. J. Virol. 88, 6611–6622 10.1128/JVI.03736-13 24696465 PMC4054376

[118] Cheng J., Gao S., Zhu C., Liu S., Li J., Kang J. et al. (2020) Typical stress granule proteins interact with the 3′ untranslated region of Enterovirus D68 to inhibit viral replication. J. Virol. 94, 10.1128/JVI.02041-19 PMC7081905 31941779

[119] Sun M., Wu S., Zhang X., Liu Z., Zhang L., Kang S. et al. (2022) Grouper TIA-1 functions as a crucial antiviral molecule against nervous necrosis virus infection. Fish Shellfish Immunol. 121, 478–486 10.1016/j.fsi.2022.01.036 35085738

[120] Wang X., Wang H., Li Y., Jin Y., Chu Y., Su A. et al. (2015) TIA-1 and TIAR interact with 5′-UTR of enterovirus 71 genome and facilitate viral replication. Biochem. Biophys. Res. Commun. 466, 254–259 10.1016/j.bbrc.2015.09.020 26363455

[121] Emara M.M., Liu H., Davis W.G. and Brinton M.A. (2008) Mutation of mapped TIA-1/TIAR Binding sites in the 3′ terminal stem-loop of west nile virus minus-strand RNA in an infectious clone negatively affects genomic RNA amplification. J. Virol. 82, 10657–10670 10.1128/JVI.00991-08 18768985 PMC2573169

[122] Emara M.M. and Brinton M.A. (2007) Interaction of TIA-1/TIAR with West Nile and dengue virus products in infected cells interferes with stress granule formation and processing body assembly. Proc. Natl. Acad. Sci. 104, 9041–9046 10.1073/pnas.0703348104 17502609 PMC1885624

[123] Gu J., Chen Z., Chen X. and Wang Z. (2020) Heterogeneous nuclear ribonucleoprotein (hnRNPL) in cancer. Clin. Chim. Acta 507, 286–294 10.1016/j.cca.2020.04.040 32376323

[124] Peddigari S., Li P.W.-L., Rabe J.L. and Martin S.L. (2012) hnRNPL and nucleolin bind LINE-1 RNA and function as host factors to modulate retrotransposition. Nucleic Acids Res. 41, 575–585 10.1093/nar/gks1075 23161687 PMC3592465

[125] Fei T., Chen Y., Xiao T., Li W., Cato L., Zhang P. et al. (2017) Genome-wide CRISPR screen identifies HNRNPL as a prostate cancer dependency regulating RNA splicing. Proc. Natl. Acad. Sci. U. S. A. 114, E5207–E5215 10.1073/pnas.1617467114 28611215 PMC5495225

[126] Sun C., Liu M., Chang J., Yang D., Zhao B., Wang H. et al. (2020) Heterogeneous nuclear ribonucleoprotein L negatively regulates foot-and-mouth disease virus replication through inhibition of viral RNA Synthesis by interacting with the internal ribosome entry site in the 5′ untranslated region. J. Virol. 94, 1–19 10.1128/JVI.00282-20 PMC7199413 32161169

[127] Zemojtel T., Kielbasa S.M., Arndt P.F., Behrens S., Bourque G. and Vingron M. (2011) CpG deamination creates transcription factor-binding sites with high efficiency. Genome Biol. Evol. 3, 1304–1311 10.1093/gbe/evr107 22016335 PMC3228489

[128] Takata M.A., Gonçalves-Carneiro D., Zang T.M., Soll S.J., York A., Blanco-Melo D. et al. (2017) CG dinucleotide suppression enables antiviral defence targeting non-self RNA. Nature 550, 124–127 10.1038/nature24039 28953888 PMC6592701

[129] Sharp C.P., Thompson B.H., Nash T.J., Diebold O., Pinto R.M., Thorley L. et al. (2023) CpG dinucleotide enrichment in the influenza A virus genome as a live attenuated vaccine development strategy. PLoS Pathog. 19, e1011357 10.1371/journal.ppat.1011357 37146066 PMC10191365

[130] Gao G., Guo X. and Goff S.P. (2002) Inhibition of retroviral RNA production by ZAP, a CCCH-type zinc finger protein. Science 297, 1703–1706 10.1126/science.1074276 12215647

[131] Ficarelli M., Neil S.J.D. and Swanson C.M. (2021) Targeted restriction of viral gene expression and replication by the ZAP antiviral system. Ann. Rev. Virol. 8, 265–283 10.1146/annurev-virology-091919-104213 34129371

[132] Xue G., Braczyk K., Gonçalves-Carneiro D., Dawidziak D.M., Sanchez K., Ong H. et al. (2022) Poly(ADP-ribose) potentiates ZAP antiviral activity. PLoS Pathog. 18, e1009202 10.1371/journal.ppat.1009202 35130321 PMC8853533

[133] Li M.M.H., Aguilar E.G., Michailidis E., Pabon J., Park P., Wu X. et al. (2019) Characterization of novel splice variants of zinc finger antiviral protein (ZAP). J. Virol. 93, e00715–e00719 10.1128/JVI.00715-19 31118263 PMC6714797

[134] Gregersen L.H., Mitter R., Ugalde A.P., Nojima T., Proudfoot N.J., Agami R. et al. (2019) SCAF4 and SCAF8, mRNA Anti-Terminator Proteins. Cell 177, 1797.e18–1813.e18 10.1016/j.cell.2019.04.038 31104839 PMC6579486

[135] Zimmer M.M., Kibe A., Rand U., Pekarek L., Ye L., Buck S. et al. (2021) The short isoform of the host antiviral protein ZAP acts as an inhibitor of SARS-CoV-2 programmed ribosomal frameshifting. Nat. Commun. 12, 7193 10.1038/s41467-021-27431-0 34893599 PMC8664833

[136] Meagher J.L., Takata M., Gonçalves-Carneiro D., Keane S.C., Rebendenne A., Ong H. et al. (2019) Structure of the zinc-finger antiviral protein in complex with RNA reveals a mechanism for selective targeting of CG-rich viral sequences. Proc. Natl. Acad. Sci. 116, 24303–24309 10.1073/pnas.1913232116 31719195 PMC6883784

[137] Luo X., Wang X., Gao Y., Zhu J., Liu S., Gao G. et al. (2020) Molecular mechanism of RNA recognition by zinc-finger antiviral protein. Cell Reports 30, 46.e4–52.e4 10.1016/j.celrep.2019.11.116 31914396

[138] Chiu H.P., Chiu H., Yang C.F., Lee Y.L., Chiu F.L., Kuo H.C. et al. (2018) Inhibition of Japanese encephalitis virus infection by the host zinc-finger antiviral protein. PLoS Pathog. 14, e1007166 10.1371/journal.ppat.1007166 30016363 PMC6049953

[139] Bick M.J., Carroll J.W., Gao G., Goff S.P., Rice C.M. and MacDonald M.R. (2003) Expression of the zinc-finger antiviral protein inhibits alphavirus replication. J. Virol. 77, 11555–11562 10.1128/JVI.77.21.11555-11562.2003 14557641 PMC229374

[140] Tang Q., Wang X. and Gao G. (2017) The short form of the zinc finger antiviral protein inhibits influenza A virus protein expression and is antagonized by the virus-encoded NS1. J. Virol. 91, 10.1128/jvi.01909–16 10.1128/JVI.01909-16 PMC5215320 27807230

[141] Galão R.P., Wilson H., Schierhorn K.L., Debeljak F., Bodmer B.S., Goldhill D. et al. (2022) TRIM25 and ZAP target the Ebola virus ribonucleoprotein complex to mediate interferon-induced restriction. PLoS Pathog. 18, e1010530 10.1371/journal.ppat.1010530 35533151 PMC9119685

[142] Zhu Y., Wang X., Goff S.P. and Gao G. (2012) Translational repression precedes and is required for ZAP-mediated mRNA decay. EMBO J. 31, 4236–4246 10.1038/emboj.2012.271 23023399 PMC3492732

[143] Li M.M.H., Lau Z., Cheung P., Aguilar E.G., Schneider W.M., Bozzacco L. et al. (2017) TRIM25 enhances the antiviral action of zinc-finger antiviral protein (ZAP). PLoS Pathog. 13, e1006145 10.1371/journal.ppat.1006145 28060952 PMC5245905

[144] Zhou H. and Costello J.C. (2017) All paths lead to TRIM25. Trends in Cancer 3, 673–675 10.1016/j.trecan.2017.08.005 28958384

[145] Zheng X., Wang X., Tu F., Wang Q., Fan Z. and Gao G. (2017) TRIM25 is required for the antiviral activity of zinc finger antiviral protein. J. Virol. 91, 1–25 10.1128/JVI.00088-17 PMC5391446 28202764

[146] Gonçalves-Carneiro D., Takata M.A., Ong H., Shilton A. and Bieniasz P.D. (2021) Origin and evolution of the zinc finger antiviral protein. PLoS Pathog. 17, e1009545 10.1371/journal.ppat.1009545 33901262 PMC8102003

[147] Ficarelli M., Wilson H., Pedro Galão R., Mazzon M., Antzin-Anduetza I., Marsh M. et al. (2019) KHNYN is essential for the zinc finger antiviral protein (ZAP) to restrict HIV-1 containing clustered CpG dinucleotides. eLife 8, e46767 10.7554/eLife.46767 31284899 PMC6615859

[148] Chen G., Guo X., Lv F., Xu Y. and Gao G. (2008) p72 DEAD box RNA helicase is required for optimal function of the zinc-finger antiviral protein. Proc. Natl. Acad. Sci. U.S.A. 105, 4352–4357 10.1073/pnas.0712276105 18334637 PMC2393818

[149] Guo X., Ma J., Sun J. and Gao G. (2007) The zinc-finger antiviral protein recruits the RNA processing exosome to degrade the target mRNA. Proc. Natl. Acad. Sci. U.S.A. 104, 151–156 10.1073/pnas.0607063104 17185417 PMC1765426

[150] Sharp C.P., Thompson B.H., Nash T.J., Diebold O., Pinto R.M., Thorley L. et al. (2023) CpG dinucleotide enrichment in the influenza A virus genome as a live attenuated vaccine development strategy. PLOS Pathogens 19, e1011357 10.1371/journal.ppat.1011357 37146066 PMC10191365

[151] Goodier J.L., Pereira G.C., Cheung L.E., Rose R.J. and Kazazian H.H. Jr (2015) The broad-spectrum antiviral protein ZAP restricts human retrotransposition. PLos Genet. 11, e1005252 10.1371/journal.pgen.1005252 26001115 PMC4441479

[152] Moldovan J.B. and Moran J.V. (2015) The zinc-finger antiviral protein ZAP Inhibits LINE and Alu Retrotransposition. PLos Genet. 11, e1005121 10.1371/journal.pgen.1005121 25951186 PMC4423928

[153] Choudhury N.R., Heikel G., Trubitsyna M., Kubik P., Nowak J.S., Webb S. et al. (2017) RNA-binding activity of TRIM25 is mediated by its PRY/SPRY domain and is required for ubiquitination. BMC Biol. 15, 105 10.1186/s12915-017-0444-9 29117863 PMC5678581

[154] Sanchez J.G., Sparrer K.M.J., Chiang C., Reis R.A., Chiang J.J., Zurenski M.A. et al. (2018) TRIM25 binds RNA to modulate cellular anti-viral defense. J. Mol. Biol. 430, 5280–5293 10.1016/j.jmb.2018.10.003 30342007 PMC6289755

[155] Gack M.U., Shin Y.C., Joo C.-H., Urano T., Liang C., Sun L. et al. (2007) TRIM25 RING-finger E3 ubiquitin ligase is essential for RIG-I-mediated antiviral activity. Nature 446, 916–920 10.1038/nature05732 17392790

[156] Cadena C., Ahmad S., Xavier A., Willemsen J., Park S., Park J.W. et al. (2019) Ubiquitin-dependent and -independent roles of E3 Ligase RIPLET in innate immunity. Cell 177, 1187.e16–1200.e16 10.1016/j.cell.2019.03.017 31006531 PMC6525047

[157] Choudhury N.R., Trus I., Heikel G., Wolczyk M., Szymanski J., Bolembach A. et al. (2022) TRIM25 inhibits influenza A virus infection, destabilizes viral mRNA, but is redundant for activating the RIG-I pathway. Nucleic Acids Res. 50, 7097–7114 10.1093/nar/gkac512 35736141 PMC9262604

[158] Liao C.-Y., Lei C.-Q. and Shu H.-B. (2021) PCBP1 modulates the innate immune response by facilitating the binding of cGAS to DNA. Cell. Mol. Immunol. 18, 2334–2343 10.1038/s41423-020-0462-3 32415261 PMC8484664

[159] Schwerk J., Soveg F.W., Ryan A.P., Thomas K.R., Hatfield L.D., Ozarkar S. et al. (2019) RNA-binding protein isoforms ZAP-S and ZAP-L have distinct antiviral and immune resolution functions. Nat. Immunol. 20, 1610–1620 10.1038/s41590-019-0527-6 31740798 PMC7240801

[160] Shaw A.E., Rihn S.J., Mollentze N., Wickenhagen A., Stewart D.G., Orton R.J. et al. (2021) The antiviral state has shaped the CpG composition of the vertebrate interferome to avoid self-targeting. PLoS Biol. 19, e3001352 10.1371/journal.pbio.3001352 34491982 PMC8423302

[161] Tuller T., Carmi A., Vestsigian K., Navon S., Dorfan Y., Zaborske J. et al. (2010) An evolutionarily conserved mechanism for controlling the efficiency of protein translation. Cell 141, 344–354 10.1016/j.cell.2010.03.031 20403328

[162] Gamble C.E., Brule C.E., Dean K.M., Fields S. and Grayhack E.J. (2016) Adjacent codons act in concert to modulate translation efficiency in yeast. Cell 166, 679–690 10.1016/j.cell.2016.05.070 27374328 PMC4967012

[163] Tesina P., Lessen L.N., Buschauer R., Cheng J., Wu C.C., Berninghausen O. et al. (2020) Molecular mechanism of translational stalling by inhibitory codon combinations and poly(A) tracts. EMBO J. 39, e103365 10.15252/embj.2019103365 31858614 PMC6996574

